# Berberine protects against diabetic kidney disease via promoting PGC‐1α‐regulated mitochondrial energy homeostasis

**DOI:** 10.1111/bph.14935

**Published:** 2020-07-07

**Authors:** Xin Qin, Ming Jiang, Yan Zhao, Jing Gong, Hao Su, Fen Yuan, Ke Fang, Xiaoyi Yuan, Xiao Yu, Hui Dong, Fuer Lu

**Affiliations:** ^1^ Institute of Integrated Traditional Chinese and Western Medicine, Tongji Hospital, Tongji Medical College Huazhong University of Science and Technology Wuhan China; ^2^ College of Pharmacy, Tongji Medical College Huazhong University of Science and Technology Wuhan China; ^3^ Department of Integrated Traditional Chinese and Western Medicine, Tongji Hospital, Tongji Medical College Huazhong University of Science and Technology Wuhan China; ^4^ Department of Urology, Tongji Hospital, Tongji Medical College Huazhong University of Science and Technology Wuhan China

## Abstract

**Background and Purpose:**

Disordered lipid metabolism and disturbed mitochondrial bioenergetics play pivotal roles in the initiation and development of diabetic kidney disease (DKD). Berberine is a plant alkaloid, used in Chinese herbal medicine. It has multiple therapeutic actions on diabetes mellitus and its complications, including regulation of glucose and lipid metabolism, improvement of insulin sensitivity, and alleviation of oxidative damage. Here, we investigated the reno‐protective effects of berberine.

**Experimental Approach:**

We used samples from DKD patients and experiments with models of DKD (db/db mice) and cultured podocytes, to characterize energy metabolism profiles using metabolomics. Molecular targets and mechanisms involved in the regulation of mitochondrial function and bioenergetics by berberine were investigated, along with its effects on metabolic alterations in DKD mice.

**Key Results:**

Metabolomic analysis suggested altered mitochondrial fuel usage and generalized mitochondrial dysfunction in patients with DKD. In db/db mice, berberine treatment reversed the disordered metabolism, podocyte damage and glomerulosclerosis. Lipid accumulation, excessive generation of mitochondrial ROS, mitochondrial dysfunction, and deficient fatty acid oxidation in DKD mouse models and in cultured podocytes were suppressed by berberine. These protective effects of berberine were accompanied by activation of the peroxisome proliferator‐activated receptor γ coactivator‐1α (PGC‐1α) signalling pathway, which promoted mitochondrial energy homeostasis and fatty acid oxidation in podocytes.

**Conclusion and Implications:**

PGC‐1α‐mediated mitochondrial bioenergetics could play a key role in lipid disorder‐induced podocyte damage and development of DKD in mice. Restoration of PGC‐1α activity and the energy homeostasis by berberine might be a potential therapeutic strategy against DKD.

## Graphical abstract

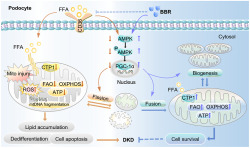

**What is already known**

- Diabetic kidney disease is one of most serious and common complications of diabetes mellitus.
- There is no effective therapy for this disease at present.

**What this study adds**

- Metabolic changes associated with diabetic kidney disease in patients, animal and cellular models were identified
- Berberine exerted therapeutic effects on the metabolic alterations in the progression of diabetic kidney disease.

**What is the clinical significance**

- Berberine might provide a new approach to the treatment of diabetic kidney disease.

## 1 INTRODUCTION

Despite strict measures aimed at improving glucose and lipid metabolism and normalizing BP, the risk of developing diabetic kidney disease (DKD) in patients with diabetes mellitus (DM) has held steady over the years (Gregg et al., 2014). Among many risk factors, lipotoxicity is generally considered as one of the main pathogenic mediators of DKD, which causes oxidative stress damage and disturbs energy homeostasis in the kidney, contributing to podocyte damage and glomerular sclerosis (Badal & Danesh, 2014; Izquierdo‐Lahuerta, Martinez‐Garcia, & Medina‐Gomez, 2016; Katsoulieris et al., 2010; Sieber & Jehle, 2014).

Kidney cells have high demands for energy to maintain their normal functions. The energy requirements of these cells are primarily satisfied by ATP generated via oxidative phosphorylation (OXPHOS) and fatty acid oxidation (FAO) contributes about 70% of the total supply (Vega, Horton, & Kelly, 2015). As the major power sources in kidney cells, mitochondria work through a set of carefully controlled gene regulation circuits (Bhargava & Schnellmann, 2017; Hock & Kralli, 2009). The peroxisome proliferator‐activated receptor (PPAR) γ coactivator‐1α (PGC‐1α) is considered to be a crucial, upstream transcriptional regulator of mitochondrial biogenesis and function (Handschin & Spiegelman, 2006; Scarpulla, 2011). This role has been demonstrated in several gain‐ and loss‐of‐function experimental studies. For example, mice lacking PGC‐1α displayed a significant reduction in oxidative metabolism and mitochondrial content (Leone et al., 2005; Tran et al., 2016). In contrast, transgenic overexpression of PGC‐1α or drug‐stimulated increase of its activity could promote mitochondrial biogenesis and FAO, increase the expression of mitochondrial genes, and inhibit kidney fibrosis and podocyte injury (Han et al., 2017; Lehman et al., 2000; Zhao et al., 2016).

Decreased PGC‐1α expression and consequent defects in mitochondrial function directly threaten cell viability, leading to cell apoptosis and dedifferentiation, thereby contributing to various metabolic diseases including diabetes, renal failure, and cardiovascular diseases (Finck & Kelly, 2006; Youle & van der Bliek, 2012). Dysfunctional mitochondria and defective FAO have been described in DKD patients and animal models (Kang et al., 2015; Li & Susztak, 2018; Mootha et al., 2003; Sharma et al., 2013). Podocytes are glomerular cells that constitute the last filtration barrier to restrict the leakage of protein into urine. Mitochondrial OXPHOS is the energy source for the central cell body of podocytes, and they mainly rely on free fatty acids (FFA) as their primary fuel source (Abe et al., 2010). However, podocytes are extremely susceptible to high levels of FFA. Enhanced FFA uptake together with a reduction in FAO and in turn intracellular lipid accumulation are detrimental to podocytes, resulting in the overproduction of mitochondrial reactive oxygen species (mitoROS), imbalance of mitochondrial dynamics and bioenergetics (Imasawa & Rossignol, 2013; Mayrhofer et al., 2009).

Therefore, the search for new compounds that would enhance FAO and protect mitochondrial function, in order to reduce lipid accumulation and metabolic disorders has become increasingly important. Many strategies and drugs with hypolipidaemic and antidiabetic effects have been shown to increase FAO by targeting the transcription of PGC‐1α (Ginsberg et al., 2010; Guo et al., 2015; Hong et al., 2014; Yuan et al., 2012). Among these, the plant‐derived alkaloid, berberine, has attracted much attention. Particularly, berberine can regulate energy metabolism by targeting PGC‐1α and this alkaloid exerted therapeutic effects in an AMP‐activated protein kinase (AMPK)‐dependent manner (Zhang et al., 2014). Here, we have explored the defective FAO and mitochondrial dysfunction mediated by dysregulated PGC‐1α, in patients and animal models with DKD. We have specifically investigated the molecular mechanisms by which berberine potently restored disturbed energy metabolism in cultured podocytes.

## 2 METHODS

### 2.1 Cell culture

Conditionally immortalized mouse podocytes (Ximbio Cat# 152136, RRID: CVCL_AS87) were obtained and cultured as previously reported (Mundel et al., 1997). Differentiated podocytes were preincubated with 0.4 μmol·L^−1^ berberine or basic medium for 12 hr. Then cells were cultured with palmitic acid (PA) for 12 hr and collected for subsequent assay.

### 2.2 RNA interference

PGC1α siRNA and control siRNA were provided by Ruibobio (Guangzhou, China) and transfected into podocytes using Liposomal Transfection Reagent (Hanheng, China) according to the manufacturer's protocol.

### 2.3 Animals

All animal care and experimental procedures conformed to the NIH Guide for the Care and Use of Laboratory Animals and were approved by the Committee for Animal Research of Huazhong University of Science and Technology (Wuhan, China). Animal studies are reported in compliance with the ARRIVE guidelines (Kilkenny et al., 2010) and with the recommendations made by the *British Journal of Pharmacology.*

Male C57BLKS/J db/db diabetic mice and their non‐diabetic littermates (7 weeks old) were purchased from the Model Animal Research Center of Nanjing University. Mice were socially housed (2‐3 mice per cage) at a constant temperature of 22°C ± 2°C, 40‐60% humidity and a 12:12 hr light/dark cycle. All animals were maintained on a normal chow diet with free access to water. The db/db mice were used as DKD models (Sharma, McCue, & Dunn, 2003) and randomly separated into three groups: db/db + vehicle, db/db + 200 mg·kg^−1^·day^−1^ of berberine (BBRL), and db/db + 300 mg·kg^−1^·day^−1^ of berberine (BBRH), with 10 mice per group. The dose of berberine was based on our previous animal studies and other research (Dong et al., 2016; Qin et al., 2019; Zhou & Zhou, 2010). Intragastric administration of berberine or vehicle was started at 7 weeks of age and maintained for 8 weeks. Body weight and blood glucose were monitored every week. At the end of the intervention, 24‐hr urine was collected and tested for microalbumin excretion. Glucose tolerance test (GTT) and insulin tolerance test (ITT) were performed after a 6‐hr fast, as reported earlier (Zhang et al., 2014). The blood glucose concentrations were measured in venous blood from the tail at 0, 15, 30, 60, 90, and 120 min after i.p. injection of glucose at 1 g·kg^−1^ or i.p. injection of insulin at 1 unit·kg^−1^, respectively. Mice were killed (overdose of pentobarbital, i.p.) and kidneys removed. Kidney samples were either fixed in 4% paraformaldehyde overnight or preserved at −80°C. Mouse glomeruli and podocyte isolation were prepared according to the protocol reported previously (Ayanga et al., 2016). At least five mice per group were examined for assessment.

### 2.4 Metabolomics

The clinical experiments were approved and supervised by the ethical review board of Tongji Medical College (#2016S192) and conformed to the international standards (US Federal Policy for the Protection of Human Subjects). The inclusion and exclusion criteria for DKD and healthy participants were in accordance with the guidelines of KDOQI (Li et al., 2017; Nelson et al., 2012). Fasting blood samples from participants were collected in EDTA‐anticoagulated tubes. The separated plasma was stored at −80°C until assay. Extraction of metabolites from plasma was performed according to the previously described method followed by a two‐step derivatization. The samples were then analysed on a TriPlus‐RSH autosampler‐Trace1300‐ISQ GC‐MS instrument (West Palm Beach, FL, USA). Parameters for sample injection, temperature programming, and MS were set according to the standard procedure (Lopez‐Bascon, Priego‐Capote, Peralbo‐Molina, Calderon‐Santiago, & Luque de Castro, 2016). Compound identification was performed using the Metabodetector software based on retention index and NIST library (version 11, 2011) searching (Hiller et al., 2009). SIMCA‐P+ software (Version 13.0, Umetrics, Umea, Sweden) was used for multivariate projection modelling and plotting.

### 2.5 Biochemical index measurements

Blood samples were obtained from participants in the morning after fasting for 12 hr and analysed by the Beckman Coulter LH 780 haematology analyser (Beckman, Coulter, FL, USA) in Tongji Hospital. Plasma FFA was measured with the FFA assay kit (Pulilai, China). Malondialdehyde (MDA) and SOD activity were assayed with the commercial kits (Nanjing Jiancheng, China). Triglyceride (TG) and hydroxynonenal (HNE) measurement in tissue extracts and cells were processed with the TG assay kit (Pulilai, China) and HNE elisa kit (Cayman, USA), respectively.

### 2.6 Human kidney tissue

The clinical study was approved by the institutional review board of Tongji Medical College (#2016S192) and conformed to the international standards (US Federal Policy for the Protection of Human Subjects). Human kidney samples were obtained from patients undergoing routine surgical nephrectomy in Tongji Hospital. Fresh tissues were fixed immediately in formalin and embedded in paraffin until use.

### 2.7 Oil Red O (ORO) staining

Kidney cryosections and podocytes were washed in 60% isopropanol and then incubated with 0.5% ORO solution (Sigma‐Aldrich) for 60 min. Samples were washed with isopropanol for 5 s and imaged.

### 2.8 Mitochondrial function assays

Podocyte mitochondria were isolated using a Mitochondria Isolation Kit (Thermo Fisher Scientific, USA). Respiratory chain complex activities, the amounts of ATP, ADP, NAD and NADH were measured using commercial kits in accordance with manufacturer's protocols.

### 2.9 mitoROS measurement

The mitoROS levels were measured using the MitoSOX Red Superoxide Indicator (Invitrogen, M36008) for 20 min at 37°C after drug intervention. Details were in accordance with the protocols (Robinson, Janes, & Beckman, 2008).

### 2.10 Western blotting

The antibody‐based procedures used in this study comply with the recommendations made by the *British Journal of Pharmacology* (Alexander et al., 2018). Western blotting was conducted as described previously (Qin et al., 2019). Antibodies used were as follows: AMPK (Cell Signaling Technology, Cat# 4811, RRID: AB_11178532), p‐AMPK (Cell Signaling Technology, Cat# 2535, RRID: AB_331250), PGC‐1α (Santa Cruz Biotechnology, Cat# sc‐518025, RRID: AB_2755043), carnitine palmitoyltransferase 1 (CPT1; Cell Signaling Technology, Cat# 12252, RRID: AB_2797857), acetyl‐CoA carboxylase (ACC) Cell Signaling Technology, Cat# 3676, RRID: AB_2219397), phosphorylate ACC (Cell Signaling Technology, Cat# 11818, RRID: AB_2687505), cluster of differentiation 36 (CD36; Novus, Cat# NB400‐144, RRID: AB_10003498), β‐actin (Cell Signaling Technology Cat# 4970, RRID: AB_2223172), anti‐rabbit secondary antibody (Cell Signaling Technology, Cat# 5151, RRID: AB_10697505), and anti‐mouse secondary antibody (Cell Signaling Technology, Cat# 5257, RRID: AB_10693543). The bands were quantified using Image J software (NIH). Original scan images are available in Supporting Information.

### 2.11 qRT‐PCR analyses

The RNA extraction, reverse transcription, and qPCR analysis were conducted as previously described (Qin et al., 2019). For mitochondrial DNA (mtDNA) copy number, cytochrome *c* oxidase subunit 4I1 (Cox4i1) was used as mtDNA marker. The relative quantity was analysed using the 2^−ΔΔCT^ method. Sequences of primers used are presented in Table S1.

### 2.12 Immunohistochemistry and immunofluorescence staining

For paraffin‐embedded samples, sections were dewaxed and rehydrated, followed by antigen retrieval, blocking with 3% H_2_O_2_ and serum. For fixed cryosections, staining was started from antigen retrieval and then blocking. Cultured cells were first fixed and blocked with serum. After that, sections or cells were incubated with antibodies. The primary antibodies used were as follows: nephrin (R&D systems, Cat# AF3159, RRID: AB_2155023), podocin (Sigma‐Aldrich, Cat# P0372, RRID: AB_261982), voltage‐dependent anion channel (VDAC; Abcam, Cat# ab14734, RRID: AB_443084), PGC‐1α (Santa Cruz Biotechnology, Cat# sc‐518025, RRID: AB_2755043), and CD36 (Novus, Cat# NB400‐144, RRID: AB_10003498). The Dako EnVision™ Detection Kit (Peroxidase/DAB, Rabbit/Mouse, K5007) was used for immunohistochemistry. Secondary anti‐rabbit, anti‐mouse, and anti‐goat antibodies were used for immunofluorescence. More than 10 fields from each sample were captured for assessment.

### 2.13 Histological analysis

For Masson's trichrome staining and periodic acid‐silver metheramine (PASM), samples were fixed in 4% paraformaldehyde overnight, and the sections were stained by Biossci Biotechnology, China.

### 2.14 Electron microscopy

Mitochondrial morphology in cultured podocytes by transmission electron microscopy (TEM) was examined as previously described (Qin et al., 2019). Conventional scanning electron microscopic (SEM) examination for mouse glomeruli was conducted according to standard protocols. In brief, small cubes of kidney cortex were fixed in 2.5% glutaraldehyde solution and immersed in 2% osmium tetroxide for 2 hr. After dehydration with gradient ethanol, samples were freeze‐dried, mounted on aluminium stubs, coated with gold and then viewed with a scanning electron microscope (Hitach su8010, Japan).

### 2.15 Data and statistical analysis

The data and statistical analysis comply with the recommendations of the *British Journal of Pharmacology* on experimental design and analysis in pharmacology (Curtis et al., 2018). Data are presented as means ± *SEM* and tested for normality via Shapiro–Wilk test, which were then tested for homogeneity of variance via one‐way ANOVA. Comparisons between two groups were performed with the Student's *t* test. And one‐way ANOVA with Tukey's test was conducted for comparisons among multiple groups. Post hoc tests were run only if *F* achieved *P* < .05, and there was no significant variance inhomogeneity. *P* values < .05 were considered statistically significant, and all tests were two‐tailed. The declared group size is the number of independent values, and the statistical analysis was done using these independent values. When outliers were included or excluded in analysis, this is stated within the figure legend. Statistics were analysed using the GraphPad Prism 6 software.

### 2.16 Materials

The compounds used in these experiments were supplied as follows: berberine from Solarbio (Beijing, China); insulin from Jiangsu Wanbang Biochemical Pharmaceutical Group (Xuzhou, China); Compound C from MedchemExpress China (Shanghai, China).

### 2.17 Nomenclature of targets and ligands

Key protein targets and ligands in this article are hyperlinked to corresponding entries in http://www.guidetopharmacology.org, the common portal for data from the IUPHAR/BPS Guide to PHARMACOLOGY (Harding et al., 2018), and are permanently archived in the Concise Guide to PHARMACOLOGY 2019/20 (Alexander, Cidlowski et al., 2019; Alexander, Fabbro et al., 2019; Alexander, Kelly et al., 2019).

## 3 RESULTS

### 3.1 Berberine improved the metabolic profiles of db/db mice and alleviated lipid accumulation in the kidney

**FIGURE 1:**
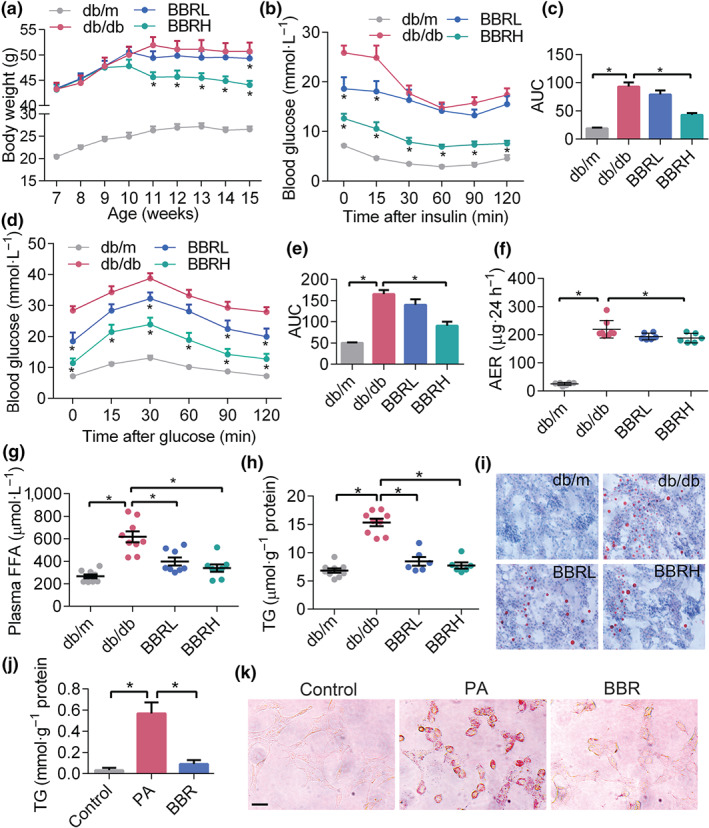
Berberine (BBR) improves the metabolic profiles of db/db mice and alleviates kidney lipid accumulation. (a) Body weight of mice during the intervention (*n* = 8). (b) Insulin tolerance test on mice injected with 1 U·kg^−1^ insulin (*n* = 8). (c) The average AUC in B (*n* = 8). (d) Glucose tolerance test on mice injected i.p. with 1 g·kg^−1^ glucose (*n* = 8). (e) The average AUC in D (*n* = 8). (f) Urinary albumin excretion of mice in 24 hr (*n* = 5). (g) Plasma FFA levels in different groups (*n* = 5). (h) TG concentration in mouse kidney (*n* = 5). (i) Kidney lipid accumulation tested by Oil Red O staining. (j) TG content in cultured podocytes treated with PA or BBR. Data were represented as mean ± *SEM*. *P < .05, significant effect of berberine or as indicated. AER, albumin excretion rate; BBRH, db/db mice treated with higher dose of BBR; BBRL, db/db mice treated with lower dose of BBR; FFA, free fatty acids; PA, palmitic acid; TG, triglyceride

The effects of berberine on metabolic alterations were tested on db/db mice. As shown in Figure 1a, the weight gain of diabetic mice was apparently controlled with 8 weeks of berberine treatment. Berberine potently improved insulin sensitivity (Figure 1b,c) and glucose tolerance (Figure 1d,e), and lowered the excretion of urinary microalbumin, as reflected by 24‐hr albumin excretion rate (AER)—the classical indicator of DKD (Figure 1f). The plasma FFA and TG content in glomeruli were also lowered by berberine treatment (Figure 1g–i). These improved metabolic conditions were particularly prominent in the group of mice treated with the higher dose of berberine. Also, in cultured podocytes treated with PA, the lipid accumulation was significantly relieved by berberine (Figure 1j,k). All these data demonstrated that berberine could, in db/db mice, improve disordered metabolic profiles, alleviate typical symptoms of DKD, and decrease lipid accumulation in the kidney.

### 3.2 Berberine reduced overproduction of ROS and podocyte injury, induced by lipid overload

**FIGURE 2:**
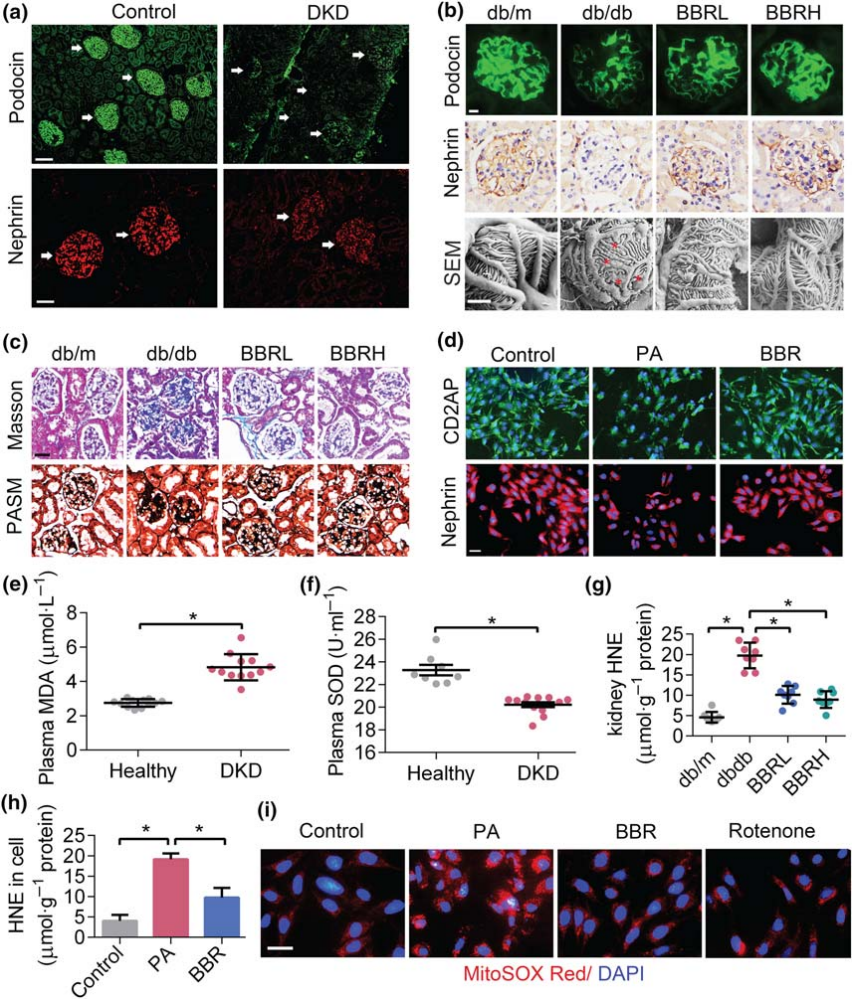
Berberine (BBR) decreases overproduction of ROS and podocyte damage. (a) Podocin and nephrin immunofluorescence in human kidney samples. White arrows denote glomeruli. Scale bars: 400 μM for row 1, 200 μM for row 2. (b) Podocin immunofluorescence, nephrin immunohistochemical staining, and SEM micrographs of mouse kidney. The red asterisks in SEM image indicate effaced podocyte foot processes. Scale bars: 50 μM for row 1 and row 2, 2 μM for row 3. (c) The Masson's trichrome staining and PASM staining. Scale bars: 100 μM. (d) CD2AP and nephrin immunofluorescence in cultured podocytes. Scale bars: 100 μM. (e, f) plasma MDA and SOD content in DKD patients and control group. (g, h) HNE levels in mouse kidney and cultured podocytes, respectively. (i) MitoSOX Red in cultured podocytes with different treatment. Scale bars: 100 μM. Data were represented as mean ± *SEM*. *P < .05, significantly different as indicated. BBRH, db/db mice treated with higher dose of BBR; BBRL, db/db mice treated with lower dose of BBR; DKD, diabetic kidney diseases; HNE, hydroxynonenal; MDA, malondialdehyde; PA, palmitic acid; ROS, reactive oxygen species; SEM, scanning electron microscope

Accumulation of FFA induces excessive ROS generation, which could then lead to lipid peroxidation, podocyte damage, and glomerulopathy. We first examined markers of podocytes, the slit diaphragm proteins (SDs) in glomeruli of DKD patients. As shown in Figure 2a, the protein levels of nephrin and podocin markedly decreased in DKD cases when compared with that of controls. Then we examined kidney samples from db/db mice and observed that berberine protected the SDs and maintained podocyte structure (Figure 2b), and ameliorated the diabetic glomerulopathy, as reflected by the thickness of glomerular basement membrane and the degree of glomerular fibrosis (Figure 2c). The protective role of berberine on podocytes in kidney tissue sections were consistent with the data from cultured podocytes (Figure 2d).

Increased levels of ROS were present in the plasma of DKD patients, as reflected by the MDA and SOD assays (Figure 2e,f). In diabetic mouse glomeruli and cultured podocytes, berberine alleviated the oxidative stress damage, as reflected by the levels of HNE (Figure 2g,h), in accordance with our previous results from DHE staining (Qin et al., 2019). Because ROS are mainly generated in the mitochondria during the process of electron transport, we assayed mtROS in PA‐induced podocytes and found mtROS production was markedly increased by PA and reduced after berberine treatment (Figure 2i). Together, these data showed that berberine could protect glomerular podocytes from FFA‐induced oxidative damage.

### 3.3 Berberine rescued mitochondrial function and improved FAO

**FIGURE 3:**
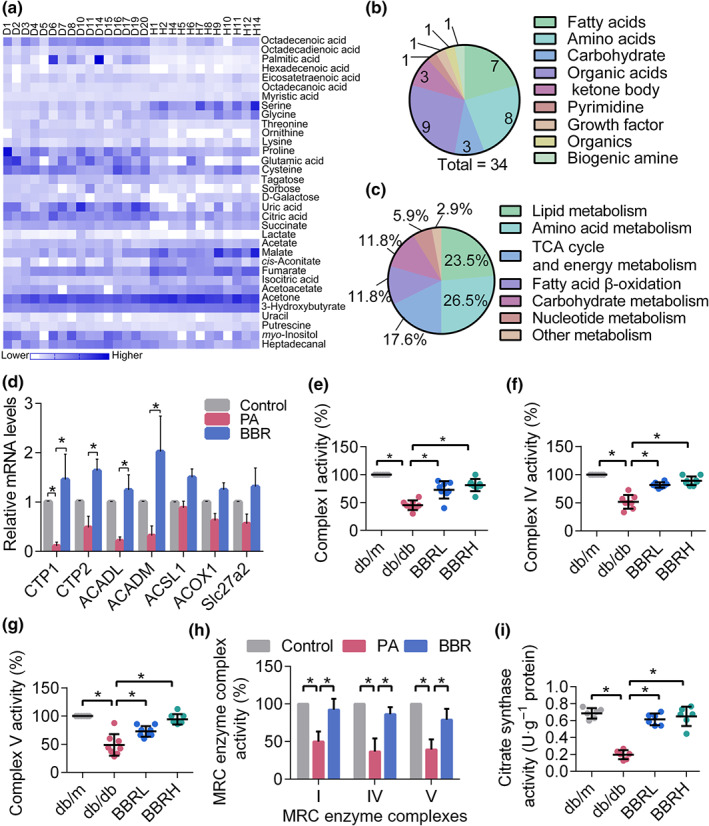
Disturbed energy homeostasis in DKD cases and the improvement of mitochondrial function and FAO by berberine (BBR). (a) Heatmap analysis for metabolites detected in DKD patients and controls. (b) Metabolites were broadly classified into different categories. (c) Alterations in metabolic pathways mainly involved. (d) Mitochondrial FAO‐related gene expression in podocytes. (e–f) Mitochondrial complex activities in mouse kidney podocytes. (h) Mitochondrial complex activities in cultured podocytes. (i) The activity of mitochondrial citrate synthetase in mouse kidney podocytes. Data were represented as mean ± *SEM*. *P < .05, significantly different as indicated. ACADL, acyl‐CoA dehydrogenase, long chain; ACADM, acyl‐CoA dehydrogenase, medium chain; ACOX1, acyl‐CoA oxidase 1; ACSL1, acyl‐CoA synthetase long‐chain family member 1; BBRH, db/db mice treated with higher dose of BBR; BBRL, db/db mice treated with lower dose of BBR; CPT1, carnitine palmitoyl transferase 1; CPT2, carnitine palmitoyl transferase 2; DKD, diabetic kidney diseases; FAO, fatty acid oxidation; MRC, mitochondrial respiratory chain; PA, palmitic acid; Slc27a2, fatty acid transporter 2

Increased ROS production is strongly associated with mitochondrial dysfunction, impaired mitochondrial OXPHOS, insufficient FAO, and disturbed energy homeostasis in DKD (Bonnard et al., 2008; Imasawa & Rossignol, 2013). In our case–control study, plasma metabolite profiles from DKD patients and healthy controls were measured using gas chromatography–MS (GC/MS). Clinical and biochemical characteristics of the participants are shown in Table S2. DKD patients have higher levels of fasting blood glucose (FBG), haemoglobin A1c (HbA1c), AER, and lower high‐density lipoproteins‐cholesterol (HDL‐C) than healthy controls. The orthogonal partial least squares discriminant analysis (OPLS‐DA) scatter plot (Figure S1) and heatmap analysis (Figure 3a) were conducted to visualize the metabolic profiles and showed a distinct segregation among the two groups. The relative abundance of plasma metabolites is shown in Table 1. A total of 106 metabolites were detected, and 27 metabolites were identified to be significantly different between cases and controls, according to the VIP analysis and *t* test analysis. Particularly, the DKD group showed higher levels of long chain fatty acids, uric acid, citric acid, succinate, and lower levels of serine, glycine, and certain tricarboxylic acid cycle (TCA) intermediates (malate, *cis*‐aconitate, fumarate, and isocitric acid). In addition, the DKD patients had significantly decreased levels of 3‐hydroxybutyrate, acetoacetate, and acetone, which indicated impaired FAO. Alterations in metabolic pathways correlated with the progression of DKD are summarized in Figure 3b,c. Most of these metabolites can be broadly classified into three pathways: lipid metabolism and fatty acid β‐oxidation, amino acid metabolism, and carbohydrate metabolism. Particularly, abnormal levels of mitochondrial metabolites involved in the TCA cycle was observed. All these data suggested altered mitochondrial fuel usage and generalized mitochondrial dysfunction in DKD patients.

**TABLE 1:** Relative abundance of plasma metabolites in patients with DKD and healthy controls

Metabolite	Compound ID	Metabolic pathway	Relative concentration		*P* value	VIP	Fold change over control	Trend
	HMDB		DKD (*n* = 22)	Control (*n* = 15)				
Octadecenoic acid	0062703	Lipid metabolism	0.666 ± 0.153	0.367 ± 0.138	<.05	2.5	1.813	Up
Octadecadienoic acid	0062784	Lipid metabolism	0.303 ± 0.057	0.208 ± 0.028	<.05	2.7	1.459	Up
Palmitic acid	0000220	Lipid metabolism	0.48 ± 0.4	0.121 ± 0.035	<.05	1.7	3.957	Up
Hexadecenoic acid	0003229	Lipid metabolism	0.02 ± 0.005	0.013 ± 0.003	<.05	1.7	1.544	Up
Eicosatetraenoic acid	0001043	Lipid metabolism	0.032 ± 0.005	0.027 ± 0.006	<.05	1	1.181	Up
Octadecanoic acid	0000827	Lipid metabolism	0.459 ± 0.067	0.409 ± 0.073	NS	1	1.123	Up
Myristic acid	0000806	Lipid metabolism	0.432 ± 0.034	0.419 ± 0.078	NS	1.2	1.031	Up
Serine	0000187	Amino acid metabolism	0.073 ± 0.015	0.162 ± 0.051	<.05	2.4	1.938	Down
Glycine	0000123	Amino acid metabolism	0.161 ± 0.05	0.29 ± 0.068	<.05	1.7	1.796	Down
Threonine	0000167	Amino acid metabolism	0.144 ± 0.042	0.112 ± 0.023	<.05	1.1	1.282	Up
Ornithine	0000214	Amino acid metabolism	0.033 ± 0.004	0.026 ± 0.003	<.05	1.1	1.28	Up
Lysine	0000182	Amino acid metabolism	0.28 ± 0.09	0.202 ± 0.082	<.05	1.2	1.383	Up
Proline	0000162	Amino acid metabolism	0.722 ± 0.257	0.507 ± 0.072	<.05	1.6	1.424	Up
Glutamic acid	0000148	Amino acid metabolism	0.038 ± 0.013	0.022 ± 0.013	<.05	1.4	1.762	Up
Cysteine	0000574	Amino acid metabolism	0.039 ± 0.008	0.03 ± 0.007	<.05	1.5	1.323	Up
Tagatose	0003418	Carbohydrate metabolism	0.078 ± 0.017	0.06 ± 0.016	<.05	1.9	1.313	Up
Sorbose	0001266	Carbohydrate metabolism	0.035 ± 0.014	0.021 ± 0.006	<.05	1.2	1.669	Up
Lactate	0000190	Carbohydrate metabolism	0.028 ± 0.017	0.024 ± 0.013	NS	1.2	1.182	Up
d‐Galactose	0000143	Carbohydrate metabolism	0.061 ± 0.012	0.06 ± 0.023	NS	1	1.012	NC
Citric acid	0000094	TCA cycle and energy metabolism	0.379 ± 0.054	0.293 ± 0.053	<.05	2.1	1.293	Up
Succinate	0000254	TCA cycle and energy metabolism	1.042 ± 0.099	0.909 ± 0.134	<.05	1.1	1.147	Up
Malate	0031518	TCA cycle and energy metabolism	0.131 ± 0.027	0.228 ± 0.061	<.05	2.4	1.668	Down
*cis*‐Aconitate	0000071	TCA cycle and energy metabolism	0.348 ± 0.282	0.549 ± 0.132	<.05	1.6	0.633	Down
Fumarate	0000134	TCA cycle and energy metabolism	0.012 ± 0.002	0.017 ± 0.003	<.05	2.2	1.281	Down
Isocitric acid	0000193	TCA cycle and energy metabolism	0.036 ± 0.006	0.051 ± 0.011	<.05	2.1	0.7	Down
Acetate	0000042	Fatty acid β‐oxidation	0.04 ± 0.006	0.048 ± 0.007	<.05	1.3	0.841	Down
Acetoacetate	0000060	Fatty acid β‐oxidation	0.11 ± 0.014	0.129 ± 0.018	<.05	1.5	0.855	Down
Acetone	0001659	Fatty acid β‐oxidation	0.54 ± 0.059	0.617 ± 0.043	NS	1.1	0.982	Down
3‐Hydroxybutyrate	0000357	Fatty acid β‐oxidation	0.125 ± 0.006	0.145 ± 0.006	<.05	1.8	1.075	Down
Putrescine	0001414	Amino acid metabolism	0.048 ± 0.01	0.048 ± 0.012	NS	1.1	1.006	NC
Uracil	0000300	Nucleotide metabolism	0.019 ± 0.005	0.019 ± 0.004	NS	1	0.999	NC
Uric acid	0000289	Nucleotide metabolism	0.162 ± 0.054	0.078 ± 0.039	<.05	1.8	2.087	Up
*myo*‐Inositol	0000211	Lipid metabolism	0.153 ± 0.036	0.122 ± 0.026	<.05	1.3	1.251	Up
Heptadecanal	0031039	Other metabolism	0.733 ± 0.138	0.541 ± 0.09	<.05	2.4	1.355	Up

Abbreviations: DKD, diabetic kidney disease; NC, no change; NS, not significant; TCA, tricarboxylic acid cycle; VIP, variable importance in projection.

Given these abnormalities in mitochondrial metabolic markers, we next assessed mitochondrial energy metabolism in mouse glomeruli and cultured podocytes. Key genes responsible for mitochondrial FAO and the activity of enzymes related with OXPHOS and TCA cycle were screened. As shown in Figure 3d–i, the expression of genes controlling β‐oxidation, the activity of complex I, IV, V involved in OXPHOS and citrate synthase involved in TCA cycle were decreased both in mitochondrial homogenates of kidneys from db/db mice and PA‐treated podocytes. Treatment of the mice with berberine normalized these variables and restored the mitochondrial energy homeostasis.

### 3.4 Berberine promoted mitochondrial biogenesis and increased energy output

**FIGURE 4:**
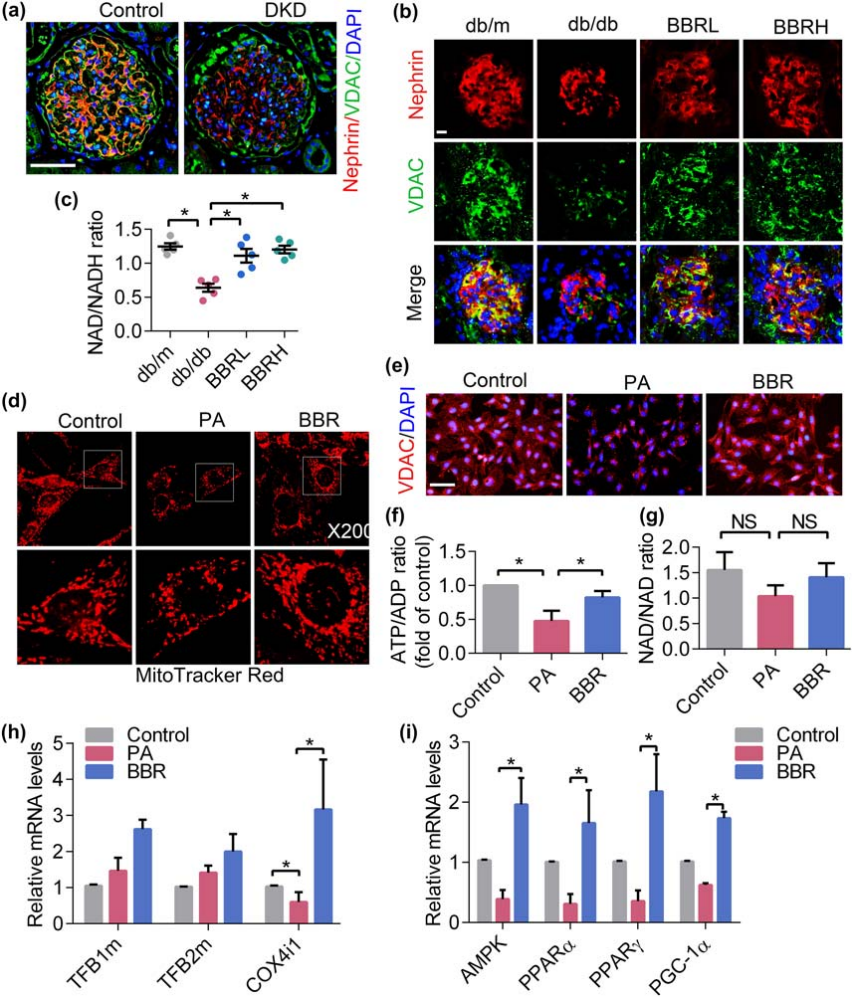
Berberine (BBR) promotes mitochondrial biogenesis and increases energy output. (a) Human kidney sections were double‐stained with nephrin and mitochondrial VDAC. nephrin, red; VDAC, green; DAPI, blue; merge, yellow. Scale bars, 200 μm. (b) Immunofluorescence of mouse kidney cryosections stained with nephrin (red) and VDAC (green). Nuclei were counterstained with DAPI (blue). Scale bars, 50 μm. (c) NAD/NADH ratios in isolated mitochondria of kidney podocytes. (d) 3D images of podocyte mitochondria stained by MitoTracker Red dye. (e) Mitochondrial shape and content were observed via VDAC staining. Scale bars, 100 μm. (f) The ATP/ADP ratio in cultured podocytes. (g) The NAD/NADH ratio in cultured podocytes. (h) The expression of mitochondrial biogenesis‐related genes was determined by RT‐PCR. (i) The gene expression of regulators of mitochondrial biogenesis in podocytes. Data shown are means ± *SEM*. *P < .05, significantly different as indicated. AMPK, AMP‐activated protein kinase; BBR, berberine; BBRH, db/db mice treated with higher dose of BBR; BBRL, db/db mice treated with lower dose of BBR; COX4i1, cytochrome c oxidase subunit 4I1; DKD, diabetic kidney diseases; NAD, nicotinamide adenine dinucleotide; NS, no significant; PGC‐1α, PPAR‐γ co‐activator 1α; TFB1m, transcription factor B1, mitochondrial; TFB2m, transcription factor B2, mitochondrial

The direct consequence of decreased mitochondrial FAO and OXPHOS activity is a reduction in energy output and mitochondrial biogenesis. To elucidate this, kidney tissue sections from DKD patients and controls were analysed with double immunofluorescence of mitochondrial VDAC and the podocyte marker, nephrin. We observed a significant reduction in podocyte numbers and mitochondria contents in glomeruli derived from DKD patients (Figure 4a). Similar results were obtained in samples from db/db mice and these changes were improved by berberine treatment (Figure 4b). Then we determined energy generation in podocytes of db/db mice as reflected by NAD^+^/NADH ratios. Our results indicated that berberine could improve the NAD^+^/NADH ratio in diabetic mouse kidneys (Figure 4c). Results from cultured podocytes also suggested that berberine protected mitochondrial biogenesis (Figure 4d,e) and increased energy generation (Figure 4f,g). In accordance with these findings, measurement of mtDNA copy number indicated a significant decrease in PA‐induced podocytes, and this was reversed by berberine intervention, as reflected by the expression of COX4i1 (Figure 4h). However, there was no significant difference in the levels of TFB1m and TFB2m. Further assessment of genes associated with both energy metabolism and mitochondrial biogenesis showed that berberine markedly increased the expression of AMPK, PPARs, and PGC1α in PA‐induced podocytes (Figure 4i).

### 3.5 Berberine regulated mitochondrial energy metabolism through AMPK/PGC1α

**FIGURE 5:**
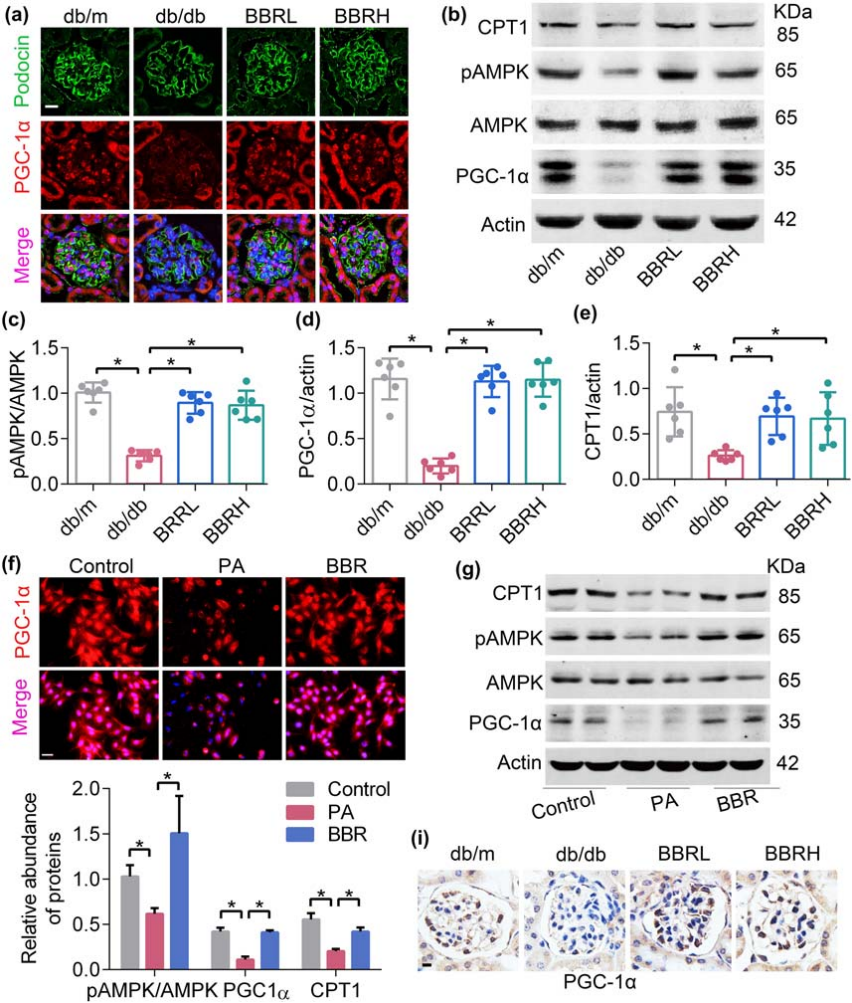
Berberine (BBR) promotes the expression of PGC‐1α in podocytes. (a) Kidney sections double‐stained with podocin and PGC‐1α. PGC‐1α, red; podocin, green; DAPI, blue; merge, pink. Scale bars, 50 μm. (b) Western blotting of AMPK, pAMPK, PGC‐1α, and CPT1 protein expression in mouse podocytes. (c–e) Quantification of protein expression shown in Figure 5b. (f) The level of PGC‐1α detected by immunofluorescence in cultured podocytes. PGC‐1α, red; DAPI, blue; merge, pink. Scale bars, 100 μm. (g) Western blotting of AMPK, pAMPK, PGC‐1α and CPT1 protein expression in cultured podocytes. (h) Quantification of protein expression shown in Figure 5h. (i) The expression of PGC‐1α detected by immunohistochemical staining in mouse glomeruli. Scale bars, 100 μm. Data shown are means ± *SEM*. *P < .05, significantly different as indicated. AMPK, AMP‐activated protein kinase; BBRH, db/db mice treated with higher dose of BBR; BBRL, db/db mice treated with lower dose of BBR; CPT1, carnitine palmitoyltransferase 1; PA, palmitic acid; PGC‐1α, PPAR‐γ co‐activator 1α

PGC‐1α controls and is controlled by several well‐known regulators involved in mitochondrial dynamics and bioenergetics (Li & Susztak, 2018). Given the profound effects of AMPK on energy metabolism, we tested whether berberine could activate AMPK, which then mediates mitochondrial energy homeostasis through activating PGC‐1α. As shown in Figure 5, berberine increased the protein level of both activated AMPK and PGC‐1α in diabetic kidneys (Figure 5a–d,i) and cultured podocytes (Figure 5f–h). Treatment with berberine also restored the expression of CPT1, the controlling enzyme for FAO (Figure 5b,e,g,h).

**FIGURE 6:**
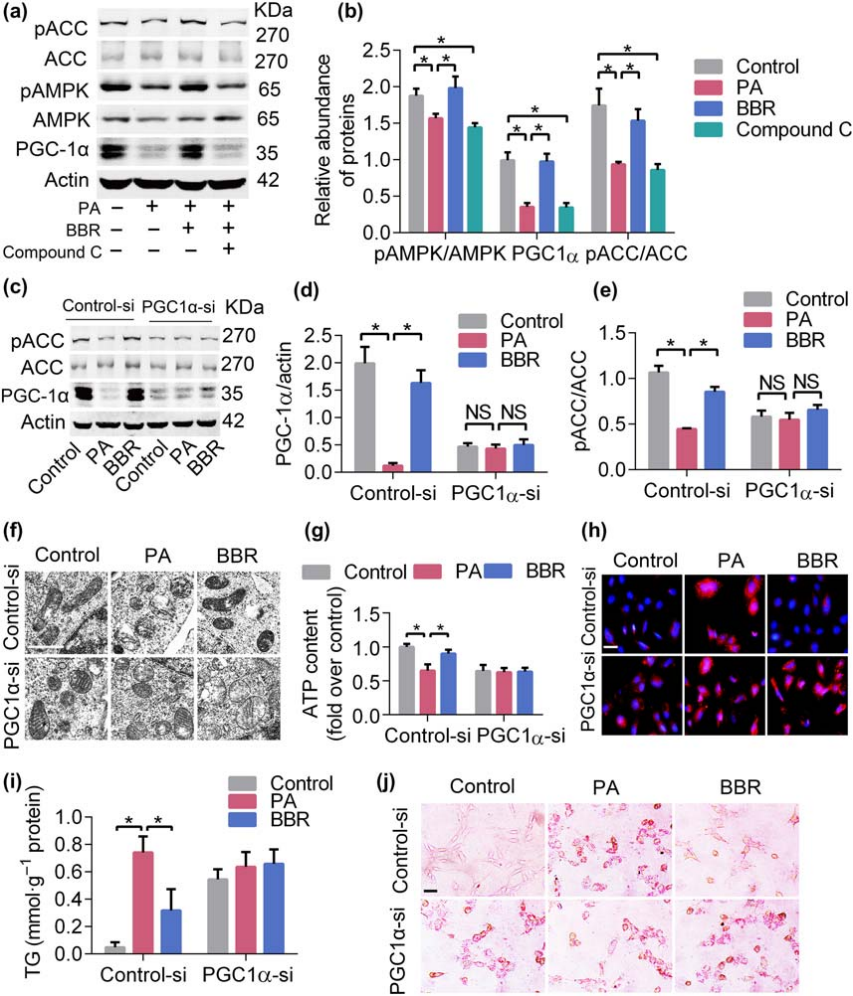
Berberine (BBR) protects mitochondrial function via the AMPK/PGC1α pathway. (a) The levels of FAO‐associated proteins after inhibition of AMPK. (b) Quantification of bands in Figure 6a. (c) Down‐regulation of PGC1α blunted the effect of BBR on FAO‐related protein expression. (d, e) Quantification of bands in Figure 6c. (f) Down‐regulation of PGC‐1α abolished the protective effects of BBR on mitochondrial morphology in podocytes. Scale bars, 1 μm. (g) The effects of BBR on ATP production was abolished after down‐regulation of PGC1α. (h) PGC1α down‐regulation increased the generation of mitoROS in podocytes. Scale bars, 100 μm. (i, j) PGC1α down‐regulation increased the lipid accumulation in podocytes. Data shown are means ± *SEM*. *P < .05, significantly different as indicated. ACC, acetyl‐CoA carboxylase; AMPK, AMP‐activated protein kinase; BBRH, db/db mice treated with higher dose of BBR; BBRL, db/db mice treated with lower dose of BBR; control‐si, control siRNA; FAO, fatty acid oxidation; PA, palmitic acid; pACC, phosphorylated acetyl‐CoA carboxylase; PGC‐1α, PPAR‐γ co‐activator 1α; PGC1α‐si, PGC‐1α siRNA; TEM, transmission electron microscopy; TG, triglyceride

We next ascertained the regulatory relationship between AMPK and PGC‐1α by using the inhibitor of AMPK, Compound C. The results showed that protein levels of PGC‐1α and phosphorylated ACC were decreased after inhibiting AMPK, and this were reversed by berberine treatment (Figure 6a,b). To further elucidate the contribution of PGC‐1α in berberine‐regulated mitochondrial function and FAO, PGC‐1α siRNA (with scrambled oligonucleotide acting as controls) was used to down‐regulate its expression. As shown in Figure 6c–j, podocytes with down‐regulated PGC‐1α were associated with reduced pACC levels, fragmentized mitochondria, increased mtROS, lower ATP content, and serious lipid accumulation. Notably, the effects of berberine on FAO and mitochondrial function were largely abolished by PGC‐1α down‐regulation. These results indicated that berberine might protect mitochondria and the related energy metabolism in podocytes, through regulating the AMPK/PGC‐1α signalling pathway.

### 3.6 Berberine inhibited FFA uptake via down‐regulating the expression of CD36

**FIGURE 7:**
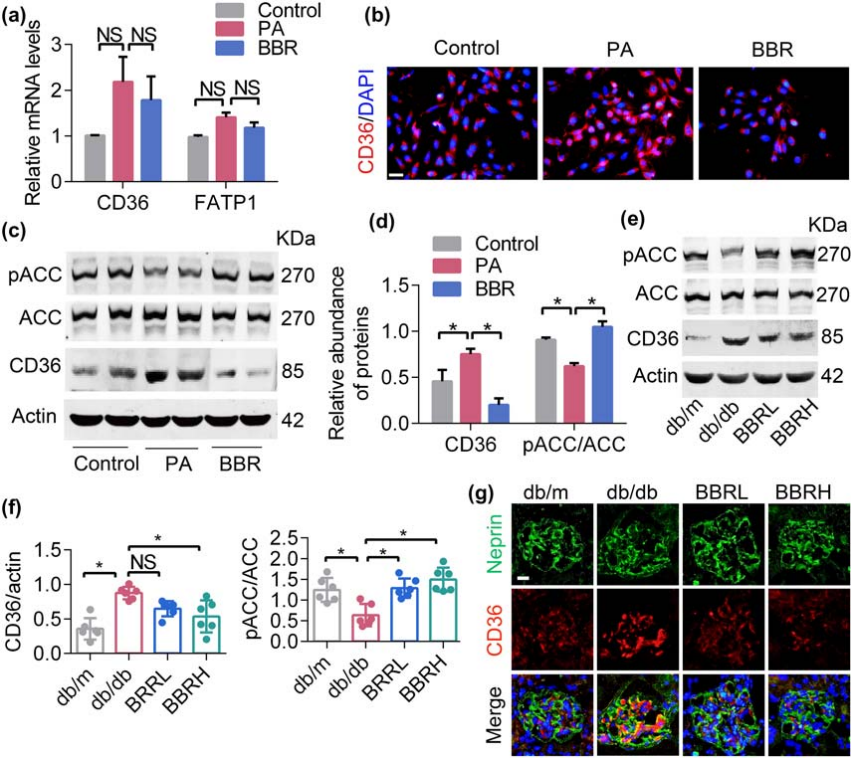
Berberine (BBR) inhibits FFA uptake via suppressing the expression of CD36. (a) FFA uptake‐related gene expression in podocytes. (b) CD36 expression in cultured podocytes examined by immunofluorescence. Scale bars, 100 μm. (c) FFA uptake and oxidation‐related protein content in cultured podocytes. (d) Quantification of bands in Figure 7c. (e) FFA uptake and oxidation‐related protein content in mouse kidney podocytes. (f) Quantification of bands in Figure 7e. (g) Mouse kidney cryosections were assessed for CD36 content via immunofluorescence. Data shown are means ± *SEM*. *P < .05, significantly different as indicated. ACC, acetyl‐CoA carboxylase; BBRH, db/db mice treated with higher dose of BBR; BBRL, db/db mice treated with lower dose of BBR; FATP1, fatty acid transport protein 1; FFA, free fatty acid; NS, not significant; PA, palmitic acid; pACC, phosphorylated acetyl‐CoA carboxylase

We next examined proteins associated with FFA uptake and synthesis. The main mediator of the cellular uptake of FFA is CD36 (scavenger receptor B2) and up‐regulation of CD36 is known to lead to lipid accumulation, increased ROS production, cell apoptosis, and kidney fibrosis (Hua et al., 2015; Yang et al., 2017). In accordance with these findings, we found that the gene expression of CD36, but not the fatty acid transport protein 1 (FATP1), was up‐regulated in PA‐induced podocytes (Figure 7a). Our results also showed that the protein levels of CD36 were markedly up‐regulated and pACC were decreased in both PA‐induced podocytes (Figure 7b–d) and db/db mice (Figure 7e–g), and this dysregulation was significantly inhibited by berberine. Collectively, our findings suggested that berberine might influence FFA metabolism and alleviate lipid accumulation partly through regulating the expression of CD36.

## 4 DISCUSSION

Our experimental and clinical studies characterized the role of mitochondrial dysfunction and disrupted FAO in the progression of DKD. We found that PGC‐1α, the master regulator of mitochondrial dynamics and energy metabolism, was significantly down‐regulated in podocytes of db/db mice, a model of DKD. PGC‐1α activation by berberine resulted in the improvement of podocyte FAO and amelioration of associated metabolic disorders in DKD mice.

Earlier studies indicated that excessive FFA and lipid accumulation could result in mitochondrial dysfunction and cell apoptosis in kidney diseases (Hua et al., 2015), whereas we still know very little about the signalling mechanisms leading to the mitochondria damage in podocytes in the development of DKD. PGC‐1α is known to be a pivotal regulator of mitochondrial homeostasis (Canto & Auwerx, 2009). Chronic metabolic disorder could disturb the activity of PGC‐1α and its downstream signalling cascades, with reduction of both FAO and energy utilization. These could then result in renal lipid overload, increased ROS production, insufficient energy supply and cell apoptosis, finally resulting in the destruction of the glomerular filtration barrier (GFB) and excessive levels of albumin in urine (Li & Susztak, 2018; Long et al., 2016; Zhu et al., 2014). Other studies have suggested that the mRNA level of PGC‐1α was significantly decreased in patients with chronic kidney disease (CKD), compared with levels in control subjects (Kang et al., 2015). Here, we have shown that cellular metabolic disorder plays a key role in podocyte damage in the development of DKD. Our results also demonstrated that dysregulated PGC‐1α, mitochondrial transcripts, and genes involved in FAO are closely linked with inefficient energy metabolism and podocyte injury.

PGC‐1α modulates mitochondrial dynamics and bioenergetics via interacting with other transcriptional factors, including PPARs and nuclear respiratory factors 1 and 2 (Canto & Auwerx, 2009; Li & Susztak, 2018). The binding of PGC‐1α to these targets will increase mitochondrial mass and global mitochondrial function, thereby enhancing FAO and energy supply (Feige & Auwerx, 2007; Vega, Huss, & Kelly, 2000). PGC‐1α also protects cells from oxidative damage via regulating the expression of several antioxidant enzymes (St‐Pierre et al., 2006). Other than that, AMPK functions as the key metabolic regulator and energy sensor (Canto & Auwerx, 2009; Dugan et al., 2013). Once activated, it can up‐regulate nutrient uptake and catabolism in response to increasing energy demand. The mechanisms by which AMPK coordinates cellular energy are reported to go through the PGC‐1α signalling pathway, either by increasing its expression or by influencing its phosphorylation (Canto & Auwerx, 2009; Jager, Handschin, St‐Pierre, & Spiegelman, 2007). AMPK could also phosphorylate ACC and increase the expression of CPT1, the master controllers of FAO, thereby promoting FAO and reducing lipid deposition (Lee et al., 2018; Zhang et al., 2014).

Previous reports indicated that improving lipid metabolism and protecting mitochondrial function by targeting PGC‐1α‐mediated mitochondrial dynamics and energy homeostasis were renoprotective in DKD models (Yuan et al., 2012). Recently, metformin was found to up‐regulate the expression of PGC‐1α, thereby tipping the balance of energy metabolism further toward higher FAO to more ATP generation (Lee et al., 2018). In podocytes, PGC‐1α activation attenuated mitochondrial dysfunction, restored expression of the SD protein and reduced cell apoptosis (Long et al., 2016; Zhou et al., 2011). Other strategies have been exploited to promote mitochondrial FAO to treat metabolic diseases either via targeting CPT1 or ACC, for example, using PPAR agonists, metformin or certain natural drug treatment, or overexpressing a CPT1 mutant to boost the activities (Dai et al., 2018; Hong et al., 2014; Lee et al., 2018). In agreement with these observations, we demonstrated that the inhibition of AMPK or PGC‐1α alone is sufficient to drive a marked reduction in FAO and to increase lipid accumulation. Our data provide further support for promoting PGC‐1α activity and FAO‐related gene expression, in order to regulate mitochondrial bioenergetics and provide benefits in DKD.

In this regard, the results with berberine showed this alkaloid to be a naturally occurring, direct activator of energy regulators, thus providing a unique pharmacological tool to promote mitochondrial energy output and FAO, specifically in kidney podocytes. Studies have previously attempted to explore the mechanisms of hypolipidemic and hypoglycaemic activity of berberine, and multiple targets and signalling pathways were observed to have transcriptional or post‐translational alterations (Ni, Ding, & Tang, 2015; Zhang et al., 2014). For instance, berberine directly stimulated the AMPK signalling pathway in fat, liver and kidney cells (Ni et al., 2015, Zhang et al., 2014). Activated AMPK could promote the phosphorylation of ACC to suppress the expression of lipid synthesis genes in cells, which significantly decreased TG and cholesterol concentration (Lee et al., 2018). Besides, berberine appears to regulate the expression of multiple genes associated with energy metabolism in mitochondria. Berberine can be as a direct activator of PGC‐1α, whose up‐regulation could induce the expression of mitochondrial and FAO and thermogenic genes (Zhang et al., 2014). Combined with previous research, our findings have strengthened the evidence for berberine as a regulator of mitochondrial function, dynamics and energy metabolism, by up‐regulating the level of PGC‐1α. These changes brought about by berberine treatment could protect renal cells from lipotoxicity, thereby decreasing extracellular matrix accumulation, alleviating glomerular sclerosis, and improving the clinical symptoms of DKD.

However, our study has some limitations. One is that failure to compare the metabolic profiles before and after berberine intervention in vivo precluded us from characterizing the role of berberine in mitochondrial function and energy metabolism, as well as other aspects of berberine's bioactivity. Another limitation is that our research could not answer whether berberine treatment could bring benefits to DKD patients, including protecting against podocyte damage, decreasing AER and restoring the GFB. These questions are of great relevance to its possible use in treating DKD in patients. We are optimistic that berberine has high potential for further exploitation because similar effects of berberine have been observed in patients with metabolic disorders such as DM. Further clinical trials and experimental research are needed to resolve these problems.

In summary, we believe that there may exist other transcription factors regulating FAO and cellular bioenergetics. Here, we have demonstrated that PGC‐1α is essential for the comprehensive regulation of energy homeostasis and might be a key target in the actions of berberine. Our present findings have revealed the important role of berberine in regulating kidney energy homeostasis and protecting glomerular podocytes from metabolic stress, and have identified berberine as a promising drug for the treatment of DKD, in the future.

## AUTHOR CONTRIBUTIONS

X.Q. designed the study, analysed the data, and drafted the manuscript; M.J. contributed to the metabolomic examination; Y.Z., J.G., and H.S. contributed to animal experiments; F.Y. and K.F. conducted cell experiments; X.Y. and X.Y. collected the human kidney samples; H.D. and F.L. carried out the clinical experiments and revised the manuscript constructively. All authors approved the final version of the manuscript.

## CONFLICT OF INTEREST

The authors declare no conflicts of interest.

## DECLARATION OF TRANSPARENCY AND SCIENTIFIC RIGOUR

This Declaration acknowledges that this paper adheres to the principles for transparent reporting and scientific rigour of preclinical research as stated in the BJP guidelines for Design & Analysis, Immunoblotting and Immunochemistry, and Animal Experimentation, and as recommended by funding agencies, publishers and other organisations engaged with supporting research.

## Supporting information

Table S1. Primers for RT‐PCR assay.
Table S2. Clinical characteristics of participants.
Figure S1. OPLS‐DA scatter plot
