# Supplementary material for: Berberine protects against diabetic kidney disease via promoting PGC‐1α‐regulated mitochondrial energy homeostasis
Source: Br J Pharmacol. 2020 Jul 7;177(16):3646–61. doi: 10.1111/bph.14935 (PMC7393204; doi:10.1111/bph.14935)
Supplement: Supplementary file 1 — Table S1. Primers for RT‐PCR assay. Table S2. Clinical characteristics of participants. Figure S1. OPLS‐DA scatter plot [file BPH-177-3646-s001.pdf]

Supporting Information

Table S1. Primers for RT-PCR assay.

Primer	Probe ID	Forward (5'→3')	Reverse (5'→3')
CPT1	NM_013495	CTACATCACCCCAACCCATATT	GATCCCAGAAGACGAATAGGTT
CPT2	NM_009949.2	TGTCTTTGATGTCCTCGATCAA	TCGGTTCTCACTGGTCAAATAA
ACADL	NM_007381.4	AAACAGTTGCACACATACAGAC	ATTCAGATGCCCAGTATTTTGC
ACADM	NM_007382.5	TCAGAGTGCCTAAGGAAAATGT	CGACTGTAGGTCTGGTTCTATC
ACSL1	NM_001302163	AATATCTACCTGCGGAGTGAAG	TTCTTCGAAGGACCCTTGTAAG
ACOX1	NM_001271898	CCAATGCTGGTATCGAAGAATG	CGACTGAACCTGGTCATAGATT
Slc27a2	NM_011978	CCCAGGATGTCATCTATACCAC	CAATGTACTGAATGACCGTGAC
TFB1m	NM_146074.1	CAGCACAGTCGCCTTTCCATT	AACAGCCTTCCAGTGCTTTCG
TFB2m	NM_001331054.1	CCAAAACCCATCCCGTCAAAT	ATGTTCCTCTGTAAGGGCTCCA
COX4i1	NM_009941.3	GGGAGTGTTGTGAAGAGTGAAGACT	TCGTGACATGGGCCACATC
AMPK	NM_001356568.1	GCCGAGAAGCAGAAGCACGAC	CACCACGTCAAGGCTCCGAATC
PPARα	NM_001113418.1	GAGCTGCAAGATTCAGAAGAAG	GAATCTTTCAGGTCGTGTTCAC
PPARγ	NM_001127330.2	CCAAGAATACCAAAGTGCGATC	TCACAAGCATGAACTCCATAGT
PGC-1α	NM_008904.2	CAACCGCAGTCGCAACATG	CCCTTTCTTGGTGGAGTGGC
CD36	NM_001159555.1	CTTTGAAAGAACTCTTGTGGGG	GTCTGTGCCATTAATCATGTCG
FATP1	NM_001357180.1	CCTCTCTGTTCTGATTCGTGTT	GTCCAGCATATACCACTACTGG
β-actin	NM_007393.3	GTGACGTTGACATCCGTAAAGA	GTAACAGTCCGCCTAGAAGCAC
18S rRNA	NR_003278	GGCGGCTTGGTGACTCTAGATAAC	CCTGCTGCCTTCCTTGGATGTG

CPT1, carnitine palmitoyltransferase 1; CPT2, carnitine palmitoyltransferase 2; ACADL, acyl-Coenzyme A dehydrogenase, long chain; ACADM, acyl-Coenzyme A dehydrogenase, medium chain; ACSL1, acyl-CoA synthetase long-chain family member 1; ACOX1, acyl-Coenzyme A oxidase 1; Slc27a2, solute carrier family 27 (fatty acid transporter), member 2; TFB1m, transcription factor B1, mitochondrial; TFB2m, transcription factor B2, mitochondrial; COX4i1, cytochrome c oxidase subunit 4I1; AMPK, AMP-activated protein kinase; PPARα, peroxisome proliferator activated receptor alpha; PPARγ, peroxisome proliferator activated receptor gamma; PGC1α, peroxisome proliferator-activated receptor-γ co-activator 1α; CD36, cluster of differentiation 36; FATP1, fatty acid transport protein 1.

Table S2. Clinical characteristics of participants.

	Health	DKD	*P* values
Number of participants (n)	15	20	
Male/female (n)	(8/7)	(9/11)	
Mean age (years)	45.87 ± 5.54	55.7 ± 8.29	<0.05
BMI（kg/m^2)	22.58 ± 3.43	23.75 ± 2.99	NS
Systolic blood pressure (mmHg)	122.6 ± 14.56	123.15 ± 9.37	NS
Diastolic blood pressure (mmHg)	76.13 ± 11.01	77.7 ± 8.97	NS
FBG (mmol/L)	5.22 ± 0.32	6.35 ± 0.82	<0.05
HbA1c (%)	5.00 ± 0.90	6.69 ± 0.92	<0.05
TC (mmol/L)	5.00 ± 0.9	4.26 ± 0.98	NS
TG (mmol/L)	1.16 ± 0.47	1.55 ± 0.83	NS
HDL-C (mmol/L)	1.45 ± 0.38	1.12 ± 0.24	<0.05
LDL-C (mmol/L)	3.04 ± 0.71	2.93 ± 1.78	NS
Urea (mmol/L)	5.17 ± 0.99	5.45 ± 1.39	NS
Serum creatinine (umol/L)	72.27 ± 14.97	74 ± 19.74	NS
eGFR (ml/min/1.73 m^2)	98.63 ± 8.23	90.33 ± 14.5	NS
AER (mg/24h)	ND	76.59 ± 40.51	

NS, no significant. ND, undetected. DKD, diabetic kidney diseases; FBG, fasting blood glucose; HbA1c, glycated hemoglobin; TC, total cholesterol; TG, triglyceride; HDL-C, high-density lipoprotein cholesterol; LDL-C, low-density lipoprotein cholesterol; eGFR, estimated glomerular filtration rate; AER, 24 h albumin excretion rati

Figure S1. OPLS-DA scatter plot

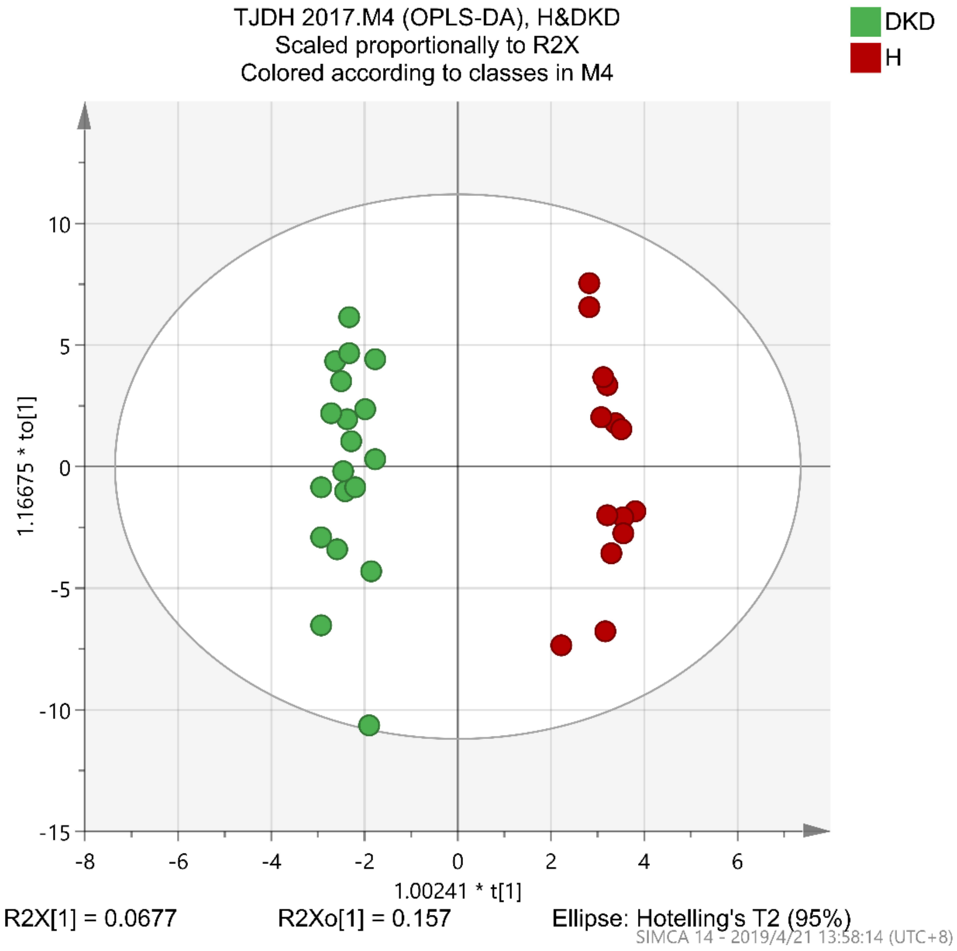

H, healthy controls; DKD, diabetic kidney diseases.
